# Supplementary material for: Nutrition in early life interacts with genetic risk to influence preadult behaviour in the Raine Study
Source: Commun Med (Lond). 2026 Feb 3;6:76. doi: 10.1038/s43856-025-01339-y (PMC12873190; doi:10.1038/s43856-025-01339-y)
Supplement: Supplementary file 2 — Supplementary Information [file 43856_2025_1339_MOESM2_ESM.pdf]

Supplementary methods:

Questionnaire formulation:

What was the last class at school that you completed?

Year _____ e.g. Year 10

OR equivalent ______________

What was your total family income before tax, per year, at the time you became pregnant?

1. Less than $7,000

2. $7,000 - $11, 999

3. $12,000 - $23,999

4. $24,000 - $35,999

5. $36,000 or more

77. Family income unknown (e.g. adolescent at home)

22. What is your martial status?

1 = Never married

2 = Married

3 = De Facto

4 = Separated or divorced

5 = Widowed

Gestational age

Gestational age was calculated from the last menstrual period or ultrasound scan at gestational week 18 ultrasound. If the discrepancy was more than 7 days the ultrasound derived measure was used.

Supplementary materials

Supplementary figure 1 – Flowchart of cohort selection:

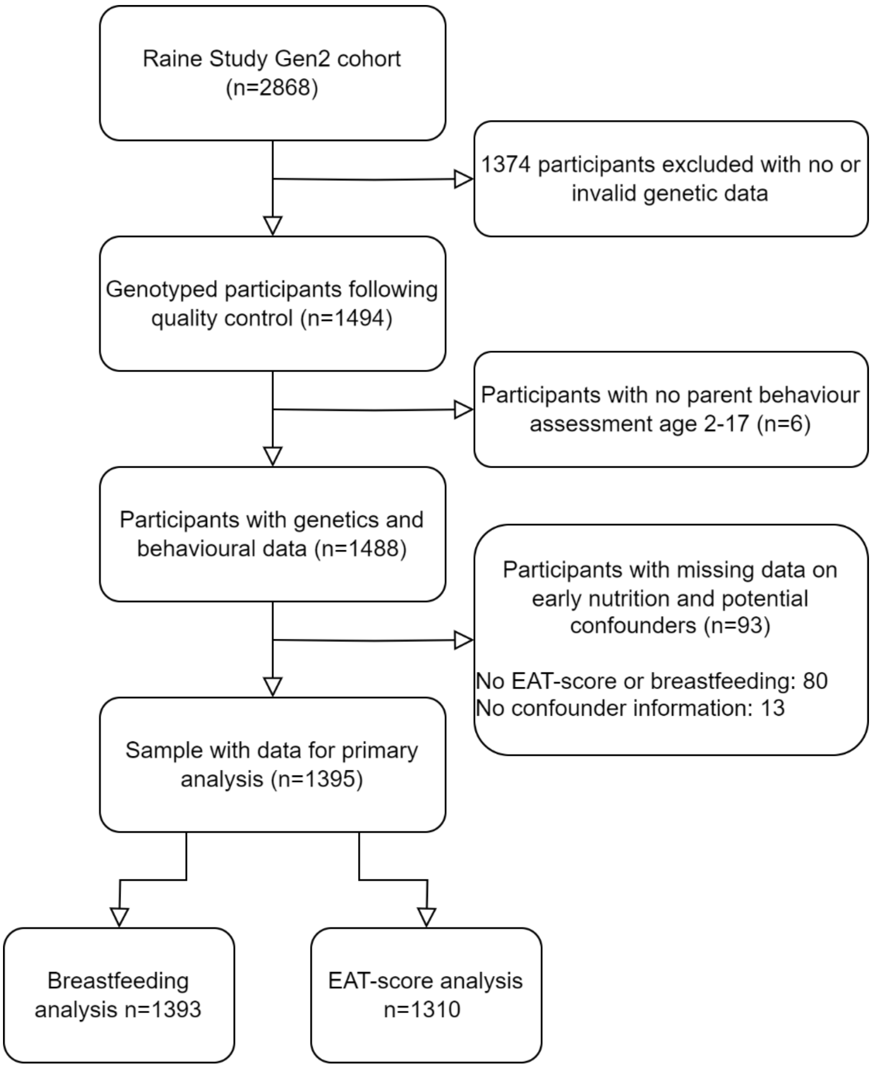

Supplementary figure 1 Flowchart of sample selection. **Gen2** = Raine Study generation 2 (offspring), **EAT** = Eating Assessment in Toddlehood,

Supplementary figure 2 – Primary variable distributions:

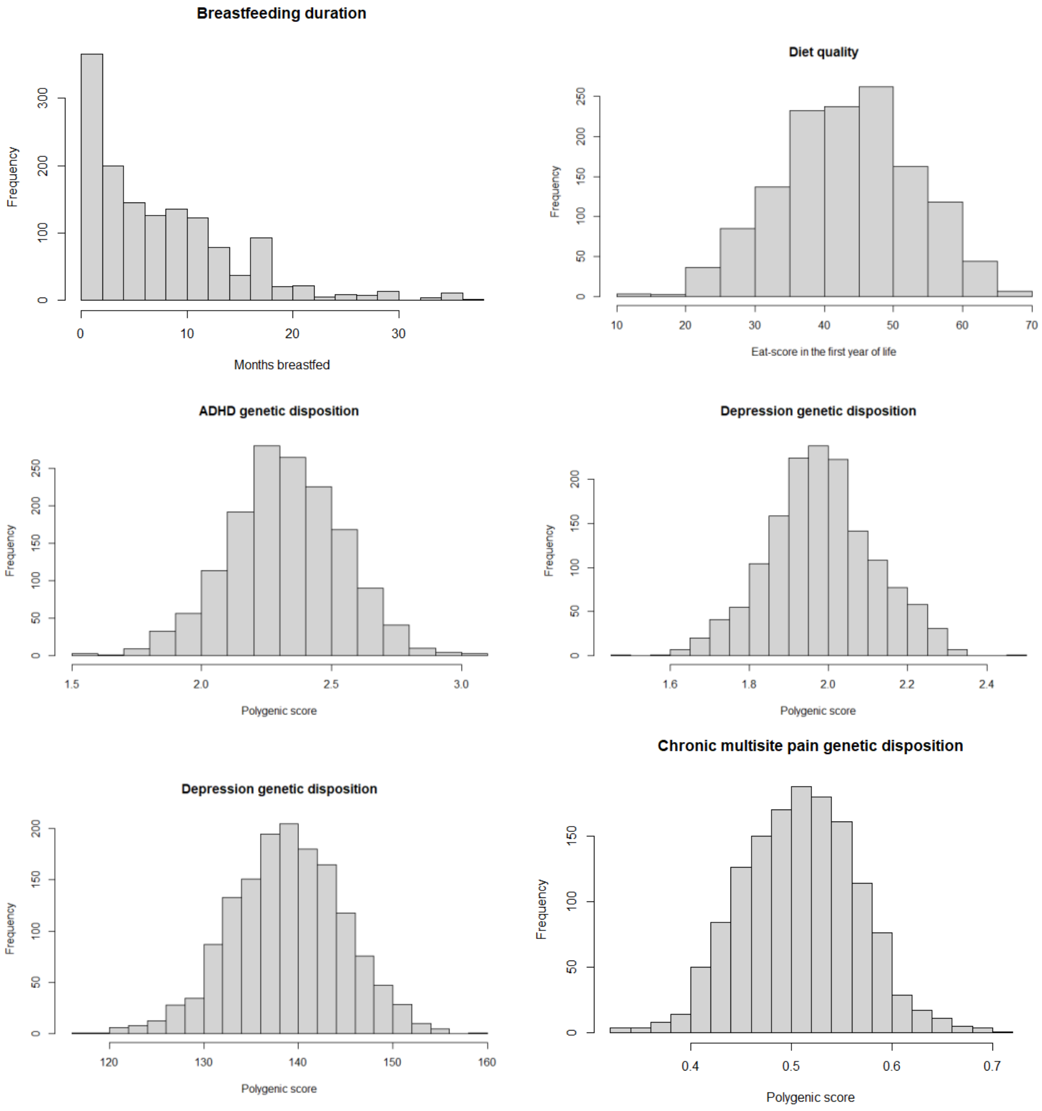

Supplementary figure 2 Histograms of primary variables. **ADHD** = Attention-deficit hyperactivity disorder.

Supplementary table 1 – Additional variables

Secondary variables for the analytic sample (n=1395)

	Breastfeeding (n=1393)	EAT year 1 (n=1310)
**Eat score age 3 (points)**	35.3 (±11.4)	35.2 (±11.4)
**Assessment age - first wave**		
Mean (SD)	2.1 (±0.1)	2.1 (±0.1)
Missing	241 (17.3%)	226 (17.2%)
**Assessment age - second wave**		
Mean (SD)	5.9 (±0.2)	5.9 (±0.2)
Missing	75 (5.5%)	71 (5.4%)
**Assessment age - third wave**		
Mean (SD)	8.1 (±0.4)	8.1 (±0.4)
Missing	87 (6.2%)	79 (6.0%)
**Assessment age - fourth wave**		
Mean (SD)	10.6 (±0.2)	10.6 (±0.2)
Missing	88 (6.3%)	80 (6.1%)
**Assessment age - fifth wave**		
Mean (SD)	14.1 (±0.2)	14.1 (±0.2)
Missing	96 (6.9%)	87 (6.6%)
**Assessment age - sixth wave**		
Mean (SD)	17.1 (±0.3)	17.1 (±0.3)
Missing	189 (13.6%)	173 (13.2%)
**Teacher score total problems age 10**		
Mean (SD)	46.3 (±10.3)	46.3 (±10.2)
Missing	169 (12.1%)	151 (11.5%)
**CBCL externalising problems age 2**		
Mean (SD)	52.1 (±8.9)	52.1 (±8.9)
Missing	295 (21.2%)	275 (21.0%)
**CBCL externalising problems age 5**		
Mean (SD)	51.7 (±10.1)	51.6 (±10.0)
Missing	106 (7.6%)	101 (7.7%)
**CBCL externalising problems age 8**		
Mean (SD)	49.6 (±10.8)	49.5 (±10.8)
Missing	126 (9.0%)	114 (8.7%)
**CBCL externalising problems age 10**		
Mean (SD)	47.3 (±10.8)	47.2 (±10.7)
Missing	106 (7.6%)	97 (7.4%)
**CBCL externalising problems age 14**		
Mean (SD)	48.1 (±11.0)	48.1 (±11.0)
Missing	133 (9.5%)	120 (9.2%)
**CBCL externalising problems age 17**		
Mean (SD)	44.9 (±10.5)	44.8 (±10.5)
Missing	395 (28.4%)	367 (28.0%)
**CBCL internalising problems age 2**		
Mean (SD)	48.8 (±9.2)	48.9 (±9.2)
Missing	295 (21.2%)	275 (21.0%)
**CBCL internalising problems age 5**		
Mean (SD)	50.0 (±10.1)	50.1 (±10.0)
Missing	106 (7.6%)	101 (7.7%)
**CBCL internalising problems age 8**		
Mean (SD)	50.4 (±10.5)	50.5 (±10.5)
Missing	126 (9.0%)	114 (8.7%)

Supplementary table 1 (continued)

**CBCL internalising problems age 10**		
Mean (SD)	49.5 (±10.6)	49.6 (±10.6)
Missing	106 (7.6%)	97 (7.4%)
**CBCL internalising problems age 14**		
Mean (SD)	46.7 (±10.7)	46.8 (±10.8)
Missing	133 (9.5%)	120 (9.2%)
**CBCL internalising problems age 17**		
Mean (SD)	44.4 (±10.3)	44.4 (±10.3)
Missing	395 (28.4%)	367 (28.0%)

Supplementary table 2 – Excluded vs analytic cohort

Comparing analytic vs excluded sample (n=2868)		
	**Excluded sample (n=1473)**	**Analytic sample (n=1395)**
**Biological sex**		
Female	740 (50.3%)	674 (48.2%)
Male	732 (49.7%)	722 (51.7%)
**Maternal education (years)**		
Mean (SD)	10.7 (±1.1)	11.0 (±1.1)
Missing	70 (4.8%)	0 (0%)
**Family Income †**		
Mean (SD)	3.4 (±1.3)	3.9 (±1.1)
Missing	174 (11.8%)	56 (4.0%)
**Civil status (Partnered)**		
Partnered	1,107 (75.2%)	1,230 (88.1%)
Single	301 (20.4%)	166 (11.9%)
Missing	64 (4.3%)	0 (0.0%)
**Maternal age (years)**		
Mean (SD)	27.1 (±6.0)	29.0 (±5.7)
Missing	72 (4.9%)	0 (0%)
**Maternal ethnicity (caucasian)**		
European descent	1,117 (75.9%)	1,355 (97.1%)
Other	291 (19.8%)	41 (2.9%)
Missing	64 (4.3%)	0 (0.0%)
**Gestation duration (weeks)**		
Mean (SD)	38.9 (±2.6)	39.4 (±2.1)
Missing	6 (0.4%)	0 (0%)
**Offspring birthweight (g)**		
Mean (SD)	3214.4 (±652.4)	3360.3 (±578.1)
Missing	10 (0.7%)	0 (0%)

†Family income had five levels: 1 Less than $7,000 2 $7,000 - $11,999 3 $12,000 - $23,999 4 $24,000 - $35,000 5 $36,000 or more

Supplementary table 3 – Baseline prediction (no interactions)

	CBCL total problems						
	**Ages 2-17***	**Age 2 (N=1135)**	**Age 5 (N=1360)**	**Age 8 (N=1340)**	**Age 10 (N=1366)**	**Age 14 (N= 1333)**	**Age 17 (N= 943)**
Own birthweight [ref]	B: 0.150<br>SE: 0.194<br>CI: [-0.239, 0.521]<br>P-value: 0.44	B: 0.218<br>SE: 0.265<br>CI: [-0.302, 0.738]<br>P-value: 0.410	B: 0.184<br>SE: 0.279<br>CI: [-0.363, 0.732]<br>P-value: 0.509	B: 0.421<br>SE: 0.304<br>CI: [-0.175, 1.018]<br>P-value: 0.166	B: 0.038<br>SE: 0.305<br>CI: [-0.561, 0.637]<br>P-value: 0.902	B: 0.033<br>SE: 0.316<br>CI: [-0.587, 0.653]<br>P-value: 0.917	B: 0.095<br>SE: 0.358<br>CI: [-0.607, 0.797]<br>P-value: 0.791
ADHD [ref]	B: 0.204<br>SE: 0.183<br>CI: [-0.151, 0.567]<br>P-value: 0.2651	**B: 0.644**<br>**SE: 0.262**<br>**CI: [0.130, 1.159]**<br>**P-value: 0.014**	B: 0.391<br>SE: 0.277<br>CI: [-0.152, 0.934]<br>P-value: 0.158	B: 0.416<br>SE: 0.304<br>CI: [-0.180, 1.012]<br>P-value: 0.171	B: 0.213<br>SE: 0.306<br>CI: [-0.387, 0.813]<br>P-value: 0.486	B: 0.003<br>SE: 0.316<br>CI: [-0.617, 0.623]<br>P-value: 0.994	B: -0.029<br>SE: 0.354<br>CI: [-0.723, 0.666]<br>P-value: 0.935
Depression [ref]	B: 0.179<br>SE: 0.187<br>CI: [-0.194, 0.538]<br>P-value: 0.34	B: 0.244<br>SE: 0.269<br>CI: [-0.284, 0.771]<br>P-value: 0.365	B: 0.135<br>SE: 0.281<br>CI: [-0.416, 0.686]<br>P-value: 0.630	B: 0.109<br>SE: 0.305<br>CI: [-0.489, 0.707]<br>P-value: 0.720	B: 0.334<br>SE: 0.306<br>CI: [-0.266, 0.934]<br>P-value: 0.275	B: 0.125<br>SE: 0.314<br>CI: [-0.491, 0.740]<br>P-value: 0.692	B: 0.147<br>SE: 0.357<br>CI: [-0.554, 0.848]<br>P-value: 0.680
Multisite chronic pain [ref]	B: 0.370<br>SE: 0.188<br>CI: [-0.003, 0.735]<br>P-value: 0.049	B: 0.159<br>SE: 0.262<br>CI: [-0.356, 0.674]<br>P-value: 0.544	B: 0.083<br>SE: 0.276<br>CI: [-0.458, 0.625]<br>P-value: 0.763	B: 0.233<br>SE: 0.301<br>CI: [-0.358, 0.823]<br>P-value: 0.440	B: 0.396<br>SE: 0.306<br>CI: [-0.205, 0.997]<br>P-value: 0.197	B: 0.617<br>SE: 0.313<br>CI: [0.003, 1.232]<br>P-value: 0.049	B: 0.315<br>SE: 0.356<br>CI: [-0.383, 1.013]<br>P-value: 0.376
Total problems [ref]	B: 0.205<br>SE: 0.38255089<br>CI: [-0.545 0.955]<br>P-value: 0.59	B: -0.069<br>SE: 0.566<br>CI: [-1.178, 1.041]<br>P-value:0.903	B: -0.099<br>SE: 0.601<br>CI: [-1.278, 1.080]<br>P-value:0.869	B: 0.397<br>SE: 0.653<br>CI: [-0.885, 1.679]<br>P-value:0.543	B: 0.182<br>SE: 0.658<br>CI: [-1.109, 1.474]<br>P-value:0.782	B: 0.381<br>SE: 0.687<br>CI: [-0.967, 1.728]<br>P-value:0.579	B: 0.711<br>SE: 0.765<br>CI: [-0.790, 2.211]<br>P-value:0.353
Breastfeeding (Months)**<br>N = 1393<br>O = 7191	B: -0.0763<br>SE: 0.0254<br>CI: [-0.125, -0.026]<br>P-value: 0.003	B: -0.094<br>SE: 0.040<br>CI: [-0.171, -0.016]<br>P-value: 0.018	B: -0.051<br>SE: 0.041<br>CI: [-0.132, 0.030]<br>P-value: 0.220	B: -0.072<br>SE: 0.045<br>CI: [-0.159, 0.016]<br>P-value: 0.109	B: -0.025<br>SE: 0.045<br>CI: [-0.114, 0.064]<br>P-value: 0.574	B: -0.126<br>SE: 0.046<br>CI: [-0.217, -0.035]<br>P-value: 0.007	B: -0.070<br>SE: 0.052<br>CI: [-0.172, 0.033]<br>P-value: 0.181
EAT1-score (0-70 points)**<br>N = 1310<br>O = 6780	B: -0.0749<br>SE: 0.020<br>CI: [-0.114, 0.036]<br>P-value: 0.0002	B: -0.095<br>SE: 0.029<br>CI: [-0.151, -0.038]<br>P-value: 0.001	B: -0.058<br>SE: 0.031<br>CI: [-0.118, 0.003]<br>P-value: 0.062	B: -0.073<br>SE: 0.033<br>CI: [-0.138, -0.007]<br>P-value: 0.029	B: -0.040<br>SE: 0.034<br>CI: [-0.106, 0.026]<br>P-value: 0.239	B: -0.096<br>SE: 0.035<br>CI: [-0.164, -0.028]<br>P-value: 0.006	B: -0.091<br>SE: 0.040<br>CI: [-0.169, -0.013]<br>P-value: 0.022

Supplementary table 3 Baseline predictive value. All models were adjusted for sex, and principal components. *Models with no nutrition had n=1395 and mixed model confidence interval (CI) and p-value in mixed models based on bootstrapped Standard Errors (SE) and two-sided z-test. ** also adjusted for age at assessment, maternal age at birth, maternal education length, family income, gestational duration, civil status.

Supplementary table 4 – Sensitivity analysis

	Age interaction	Rating age two	T-score >=60 **	Teacher rating	Term Born only
	**Sensitivity diet effects by genetic ADHD risk**				
	N = 1310, O = 6780	N=1035	N = 1310, O = 6780	N=1159	N=1205, O= 6247
Age 1 EAT-score (points)<br>Low genetic risk	B: -0.00440<br>SE: 0.0031<br>CI: [-0.0104, 0.00157]<br>P-value: 0.15	**B: -0.098**<br>**SE: 0.036**<br>**CI: [-0.169, -0.027]**<br>**P-value: 0.007**	OR: 0.752<br>SE: 0.088<br>CI: [0.597, 0.946]<br>P-value: 0.015	B: -0.041<br>SE: 0.041<br>CI: [-0.121, 0.039]<br>P-value: 0.311	B: -0.114<br>SE: 0.0263<br>CI: [-0.165, -0.0623]<br>P-value: 0.00001
Age 1 EAT-score (points)<br>High genetic risk	B: 0.000738<br>SE: 0.004<br>CI: [-0.0071, 0.0087]<br>P-value: 0.86	B: -0.087<br>SE: 0.047<br>CI: [-0.180, 0.005]<br>P-value: 0.065	OR: 1.021<br>SE: 0.140<br>CI: [0.780, 1.336]<br>P-value: 0.881	B: 0.033<br>SE: 0.050<br>CI: [-0.065, 0.130]<br>P-value: 0.507	B: 0.000580<br>SE: 0.0323<br>CI: [-0.0633, 0.0632]<br>P-value: 0.99
Interaction<br>EAT score by ADHD-PGS	B: 0.00125<br>SE: 0.002466446<br>CI: [-0.00364, 0.006]<br>P-value: 0.61	B: 0.026<br>SE: 0.026<br>CI: [-0.025, 0.077]<br>P-value: 0.320	OR: 1.167<br>SE: 0.094<br>CI: [0.996, 1.367]<br>P-value: 0.057	B: 0.014<br>SE: 0.029<br>CI: [-0.043, 0.071]<br>P-value: 0.630	B: 0.0594<br>SE: 0.0205<br>CI: [0.0187, 0.0989]<br>P-value: 0.004
	**Sensitivity breastfeeding effects by genetic chronic multisite pain risk**				
	N = 1393, O = 7191	N=1098	N = 1393, O = 7191	N = 1224	N = 1281, O = 6626
Breastfeeding (Months)<br>Low genetic risk	B: 0.00107<br>SE: 0.0041<br>CI: [-0.007, 0.0091]<br>P-value: 0.79	B: -0.083<br>SE: 0.057<br>CI: [-0.195, 0.029]<br>P-value: 0.144	OR: 0.845<br>SE: 0.150<br>CI: [0.597, 1.196]<br>P-value: 0.341	B: -0.065<br>SE: 0.059<br>CI: [-0.181, 0.052]<br>P-value: 0.275	B: -0.0241<br>SE: 0.0373<br>CI: [-0.0980, 0.0484]<br>P-value: 0.52
Breastfeeding (Months)<br>High genetic risk	B: -0.00550<br>SE: 0.0045<br>CI: [-0.0142, 0.0036]<br>P-value: 0.23	B: -0.112<br>SE: 0.055<br>CI: [-0.221, -0.003]<br>P-value: 0.044	OR: 0.807<br>SE: 0.137<br>CI: [0.578, 1.127]<br>P-value: 0.208	B: -0.040<br>SE: 0.061<br>CI: [-0.160, 0.079]<br>P-value: 0.507	B: -0.125<br>SE: 0.0423<br>CI: [-0.208, -0.0420]<br>P-value: 0.003
Interaction<br>Breastfeeding by CMSP-PGS	B: -0.00263<br>SE: 0.003<br>CI: [-0.0088, 0.004]<br>P-value: 0.41	B: -0.028<br>SE: 0.037<br>CI: [-0.100, 0.045]<br>P-value: 0.451	OR: 0.890<br>SE: 0.105<br>CI: [0.707,1.121]<br>P-value: 0.322	B: -0.045<br>SE: 0.041<br>CI: [-0.126, 0.036]<br>P-value: 0.277	B: -0.0762<br>SE: 0.023<br>CI: [-0.133, -0.0165]<br>P-value: 0.01

Supplementary table 4: Adjusted for gestational duration, maternal age, years of schooling completed, family income and civil status, offspring sex, age at assessment and principal components. Confidence interval (CI) and p-value in mixed models based on bootstrapped Standard Errors (SE) and two-sided z-test. ** Due to convergence issues the EAT1 score was scaled (subtracted mean and divided SD).

Supplementary table 5 – Sex stratified models for the BW-PGS

**Total problems**				
	**Breastfeeding**		**EAT score year 1**	
	**Males,** N= 721, O=3714	**Females,** N= 672,O=3477	**Males,** N=672, O=3470	**Females,** N=638,O=3310
Nutrition measure	B: -0.0653<br>SE: 0.0383<br>CI: [-0.141, 0.00959]<br>P-value: 0.09	B: -0.0921<br>SE: 0.03670<br>CI: [-0.164, -0.0198]<br>P-value: 0.012	B: -0.0206<br>SE: 0.0266<br>CI: [-0.0722, 0.0322]<br>P-value: 0.44	B: -0.136<br>SE: 0.02934742<br>CI: [-0.195, -0.0798]<br>P-value: 0.000004
Genetic risk (SD)	B: -0.685<br>SE: 0.4083<br>CI: [-1.48, 0.116]<br>P-value: 0.09	B: 0.543<br>SE: 0.3954<br>CI: [-0.245, 1.30]<br>P-value: 0.17	B: 1.90<br>SE: 1.1216<br>CI: [-0.317, 4.08]<br>P-value: 0.09	B: 0.331<br>SE: 1.2333<br>CI: [-2.11, 2.72]<br>P-value: 0.79
Interaction	B: 0.0521<br>SE: 0.0365<br>CI: [-0.0197, 0.123]<br>P-value: 0.15	B: -0.00673<br>SE: 0.0344<br>CI: [-0.0740, 0.0608]<br>P-value: 0.84	B: -0.0513<br>SE: 0.0254<br>CI: [-0.101, -0.00118]<br>P-value: 0.04	B: 0.00714<br>SE: 0.0279<br>CI: [-0.0471, 0.0621]<br>P-value: 0.8

Supplementary table 5 Exploring sex differences in BW-PGS effects. All models adjusted for sex, age at assessment, maternal age at birth, maternal education length, family income, gestational duration, civil status and principal components. Confidence interval (CI) and p-value in mixed models based on bootstrapped Standard Errors (SE) and two-sided z-test.

Supplementary table 6 – Exploring family income missing values

	**Missing Family income assigned highest value (=5)**	**Missing Family income assigned lowest value (=1)**
Breastfeeding * CMSP PGS<br>N = 1393, O = 7191	B: -0.0602<br>SE: 0.0277<br>CI: [-0.112, -0.00365]<br>P-value: 0.03	B: -0.0593<br>SE: 0.0277<br>CI: [-0.112, -0.00361]<br>P-value: 0.03
EAT-score * ADHD PGS<br>N = 1310, O = 6780	B: 0.0642<br>SE: 0.0178<br>CI: [0.0286, 0.0982]<br>P-value: 0.0003	B: 0.0629<br>SE: 0.0180<br>CI: [0.0269, 0.0975]<br>P-value: 0.0005

Supplementary table 6 Exploring the impact of missing family income values. All models adjusted for sex, age at assessment, maternal age at birth, maternal education length, gestational duration, civil status and principal components. Confidence interval (CI) and p-value in mixed models based on bootstrapped Standard Errors (SE) and two-sided z-test.

Supplementary table 7 individual plant-based food items:

	**Higher ADHD PGS**<br>**Age 1** estimates<br>N=516, O= 2666	**Higher ADHD PGS**<br>**Age 3** estimates<br>N=516, O= 2666	**Lower ADHD PGS**<br>**Age 1** estimates<br>N=794, O=4114	**Lower ADHD PGS**<br>**Age 3** estimates<br>N=794, O=4114
Wholegrain	B: 0.000925<br>SE: 0.10392841<br>CI: [-0.201, 0.207]<br>P-value: 0.99	B: -0.141<br>SE: 0.10193666<br>CI: [-0.343, 0.0563]<br>P-value: 0.17	B: -0.246<br>SE: 0.08243294<br>CI: [-0.404, -0.0811]<br>P-value: 0.003	B: -0.150<br>SE: 0.08221277<br>CI: [-0.307, 0.0152]<br>P-value: 0.07
Vegetables	B: -0.0965<br>SE: 0.08171734<br>CI: [-0.257, 0.0636]<br>P-value: 0.24	B: -0.0698<br>SE: 0.08573953<br>CI: [-0.238, 0.0980]<br>P-value: 0.42	B: -0.114<br>SE: 0.06779293<br>CI: [-0.244, 0.0214]<br>P-value: 0.09	B: -0.140<br>SE: 0.06736347<br>CI: [-0.271, -0.00744]<br>P-value: 0.04
Fruits	B: 0.116<br>SE: 0.08581843<br>CI: [-0.0549, 0.282]<br>P-value: 0.18	B: 0.0213<br>SE: 0.09128597<br>CI: [-0.157, 0.201]<br>P-value: 0.82	B: -0.169<br>SE: 0.07402488<br>CI: [-0.315, -0.0248]<br>P-value: 0.022	B: -0.150<br>SE: 0.07505048<br>CI: [-0.307, 0.0152]<br>P-value: 0.05

Supplementary table 7 Sub-categories of plant-based food items. All models adjusted for sex, age at assessment, maternal age at birth, maternal education length, gestational duration, civil status and principal components. Confidence interval (CI) and p-value in mixed models based on bootstrapped Standard Errors (SE) and two-sided z-test.
